# Content-function word production in Mandarin-speaking Broca’s aphasia: a generative approach

**DOI:** 10.3389/fnins.2026.1738973

**Published:** 2026-04-02

**Authors:** Shengnan Ma, Hui Chang, Yanling Xi, Mengmeng Hu

**Affiliations:** 1 School of Foreign Languages, Shanghai Jiao Tong University, Shanghai, China; 2 National Research Centre for Language and Well-being, Shanghai, China; 3 Shanghai Pudong New Area Guangming Hospital of Traditional Chinese Medicine, Shanghai, China; 4 School of Humanities, Shanghai Jiao Tong University, Shanghai, China

**Keywords:** adaptation theory, Broca’s aphasia, production, Tree-Pruning Hypothesis, word categories

## Abstract

**Introduction:**

Broca’s aphasia is a non-fluent language disorder typically associated with reduced grammatical encoding and relatively preserved lexical meaning. While content-function asymmetries have been extensively documented for Indo-European languages, corresponding evidence from Mandarin remains limited, despite Mandarin’s typological profile as an isolating, topic-prominent language whose grammatical relations are largely expressed through word order and functional particles rather than inflections.

**Methods:**

We analyzed picture-description discourse (Western Aphasia Battery picnic scene) from 18 Mandarin-speaking individuals with Broca’s aphasia and 18 healthy controls. Transcripts were segmented at the morpheme level and coded into lexical versus functional categories and their subtypes under a generative architecture.

**Results:**

Relative to the healthy controls, the patients showed a markedly pronounced content–function imbalance, producing much higher proportions of content morphemes than function morphemes in connected speech. The patients produced substantially more content items than function ones; within lexical categories, nouns constituted the largest lexical category, whereas verb proportions did not differ significantly between groups in the present sample. Within the functional domain, elements plausibly associated with lower nominal structure (e.g., demonstratives and classifiers) were sporadically available, while categories linked to higher clausal and discourse-related structure (e.g., complementizer- and topic-related markers) were near-absent.

**Discussion:**

Overall, the results indicate a robust content function imbalance in Mandarin Broca’s aphasia and motivate further individual-level work on which functional categories are most vulnerable, with potential implications for assessment and rehabilitation targets.

## 1 Introduction

Aphasia refers to a set of acquired deficits in both receptive and expressive language abilities, typically resulting from acute or progressive neurological conditions, such as a stroke affecting the left middle cerebral artery (Stefaniak et al., 2019). Impaired lexical retrieval can significantly hinder semantically coherent narrative (MacWhinney et al., 2010) and the discourse construction of people with aphasia (Kintz et al., 2016). Meanwhile, omitted or substituted function words (e.g., pronouns, conjunctions) frequently reported in the literature (Kemmerer, 2014; Goodenough et al., 1977; Butterworth, 1994; Bastiaanse and Van Zonneveld, 2005) always lead to speech characterized by reduced syntactic complexity and over-reliance on content words, ultimately constraining both sentence formulation and overall discourse organization. Therefore, a phenomenon worth exploring in aphasia research is the differential impairment of word categories, that is, function words are disproportionately affected compared to content words (Kemmerer, 2014). This imbalance is particularly salient in Broca’s aphasia, where speech is marked by a predominance of content words and omission of function words (Cui, 2018).

Broca’s aphasia is commonly described as a non-fluent aphasia syndrome characterized by effortful, reduced speech output and relatively preserved comprehension, although substantial variability exists across individuals (Goodglass et al., 1993). In connected speech, a hallmark pattern is the reduction or omission of closed-class/functional material (e.g., pronouns, particles, conjunction-like elements), which yields reduced syntactic complexity and an over-reliance on semantically rich lexical items (Kemmerer, 2014; Goodenough et al., 1977; Butterworth, 1994; Bastiaanse and Van Zonneveld, 2005). Importantly, this study does not assume a categorical dissociation between aphasia subtypes; rather, we focus on a WAB-classified Broca’s aphasia cohort as a principled test case for examining functional-structure vulnerability in non-fluent production. Consistent with this focus, recent discourse work has shown that individuals with non-fluent aphasia tend to produce fewer core function words in narrative discourse than other groups, highlighting function-word measures as a sensitive index of grammatical scaffolding in spontaneous production (Kim et al., 2021).

Although a substantial literature has investigated lexical and functional category production in aphasia, the cross-linguistic evidence base remains uneven and is strongly skewed toward a small set of widely studied languages, particularly English (Beveridge and Bak, 2011; Bates et al., 1991). This imbalance matters because languages differ in how grammatical relations and discourse packaging are overtly realized. Mandarin is especially informative in this respect: it is commonly characterized as an isolating and topic-prominent language with minimal inflectional morphology, where grammatical relations are primarily expressed through constituent order and functional particles within the nominal and clausal domains (Huang et al., 2009; Li and Thompson, 1981; Lin, 2006; Shi, 2000; Xu and Langendoen, 1985). In Mandarin, classifiers are nominal functional morphemes that typically occur in numeral/demonstrative-noun phrases and encode noun-class information (e.g., yi zhi gou “one CL dog”). Therefore, Mandarin provides a stringent test case for accounts of grammatical impairment that make claims about vulnerability of functional structure, because “functional morphology” in Mandarin is realized largely through independent particles and configurations rather than overt agreement/tense inflections.

Existing work on Chinese aphasia has reported content–function imbalances and several language-relevant phenomena such as classifier production and noun–verb dissociations (e.g., Tzeng et al., 1991; Chen and Bates, 1998; Li and Kiran, 2024). At the discourse level, recent Mandarin AphasiaBank studies and WAB picture-description work have quantified lexical retrieval and global microstructural indices (e.g., core lexicon measures, MLU, CIU-related metrics), providing valuable benchmarks for Mandarin narrative performance (Jiang et al., 2023; Deng et al., 2025). However, compared with this growing discourse literature, connected-speech evidence that systematically profiles multiple functional morpheme subtypes in Mandarin, and evaluates whether the reduction pattern is graded across lower versus higher functional/discourse layers, remains limited. In this paper, “hierarchical distribution of function-word subtypes” refers to whether functional morphemes associated with relatively lower structural domains (e.g., nominal functional material such as classifiers/demonstratives; low clausal operators such as aspect/negation) are more available than morphemes associated with the left-peripheral/discourse domain (e.g., clause-typing particles and topic-related markers), as predicted by hierarchy-based accounts of functional-structure vulnerability. The present study addresses this gap by analyzing not only the overall content–function imbalance but also the hierarchical distribution of function-word subtypes in Mandarin Broca’s aphasia.

At the same time, Mandarin’s grammar makes it essential to interpret function-word reduction against a typologically appropriate baseline: because Mandarin lacks articles and many function elements are optional or discourse-conditioned, function-word proportions cannot be assumed to pattern like English by default (Lin, 2006; Li and Thompson, 1981). A careful subtype-based analysis in naturalistic discourse, complemented by benchmark comparisons to reference Mandarin picture-description data, is therefore needed to establish whether Mandarin Broca’s aphasia shows a selective vulnerability of functional structure over and above the language’s baseline distribution.

To address these gaps, the present study investigates content and function morpheme production and their subtypes in WAB picture-description discourse from Mandarin-speaking individuals with Broca’s aphasia. We adopt a focused theoretical lens, i.e., we treat the Tree-Pruning Hypothesis (Friedmann and Grodzinsky, 1997) as the primary structural account of selective impairment in the functional domain and use a adaptation perspective (e.g., Kolk and Heeschen, 1992; Kolk, 1998) as a complementary mechanism to explain why functional encoding may be especially fragile in spontaneous production. This approach allows us to link quantitative category profiles to testable predictions about which portions of the functional hierarchy are most vulnerable in a typologically distinct language.

## 2 Theoretical foundation

### 2.1 Content vs. function words

In this study, the content–function distinction is treated as an operational, grammar-based contrast grounded in generative syntax. Specifically, content (lexical) items are those associated with lexical projections such as NP/VP/AP and typically carry core conceptual meaning (e.g., nouns, verbs, adjectives), whereas function items are closed-class morphemes associated with functional projections and heads that encode grammatical relations and discourse packaging (e.g., determiner-/classifier-related material in the nominal domain; aspect/negation and clause-typing/topic-related particles in the clausal/discourse domain). This operational definition is the basis for our morpheme-level coding and statistical analyses.

Descriptive characterizations of content versus function words often note that lexical items are semantically richer and more referential, whereas function morphemes are more abstract and serve primarily grammatical roles (e.g., Bird et al., 2002; Harley, 2006). While such semantic descriptions are useful for broad orientation, they do not constitute the theoretical definition adopted here. Rather, our classification follows a generative architecture in which lexical and functional elements differ in their structural distribution and in the type of grammatical information they encode, allowing us to test whether functional structure is selectively vulnerable in Mandarin non-fluent aphasia.

### 2.2 Vulnerability of functional structure in Mandarin

To explain content-function asymmetries in non-fluent aphasia, we consider two influential but theoretically distinct approaches: a structure-based generative account and a processing/resource-based adaptation account. The Tree-Pruning Hypothesis (TPH) proposes that impairment targets functional structure, such that projections above a damaged node are unavailable while lower projections remain relatively accessible, predicting a graded vulnerability within the functional domain (Friedmann and Grodzinsky, 1997). In contrast, adaptation-based approaches explain grammatical reduction as a consequence of limited processing resources: speakers preferentially produce forms that maximize communicative payoff and minimize planning and integration cost, which can yield function-word reduction without positing a structurally pruned tree (Kolk and Heeschen, 1992; Kolk, 1998). In this study, we do not treat these accounts as interchangeable; instead, we specify predictions that can be evaluated using Mandarin functional morphemes in connected speech.

Mandarin is typologically informative because functional distinctions are largely realized through independent particles and nominal functional structure rather than obligatory inflectional tense/agreement. In the nominal domain, demonstratives and classifiers instantiate articulated functional layers within DP/NumP/ClP (Cheng and Sybesma, 1999; Huang et al., 2009). In the clausal domain, temporal interpretation is widely argued to be derived from aspectual particles, temporal adverbials, and discourse inference rather than morphological tense, and Mandarin is often analyzed as “tenseless” in the relevant grammatical sense (Lin, 2006). Mandarin also exhibits a rich left-peripheral/discourse organization associated with topic–comment structure and topic chains (Li and Thompson, 1976; Shi, 2000; Xu and Langendoen, 1985). These properties allow us to test hierarchy-related vulnerability in a language where “functional morphology” is overtly realized mainly through particles and nominal functional heads.

Based on these considerations, we derive two sets of predictions. Under a hierarchy-based vulnerability account (TPH), functional morphemes associated with lower structural domains should be relatively more available than those associated with higher clausal/discourse structure. Operationally, we treat nominal functional material (e.g., determiners/demonstratives and classifiers) and low clausal operators (e.g., aspect markers and negation) as comparatively lower, whereas left-peripheral/discourse-related devices (e.g., clause-typing particles and topic-related markers) are treated as higher. Accordingly, TPH predicts a graded pattern in which higher/discourse-related functional types are disproportionately reduced relative to lower functional types in the Broca’s aphasia group. In contrast, adaptation-based accounts predict that retention versus omission is better explained by frequency, salience, and processing cost: highly frequent and low-burden forms may be preserved even when they are analyzed high, whereas less frequent or planning-intensive forms may be reduced regardless of projection height. Therefore, a pattern tightly tracking structural “height” favors hierarchy-based vulnerability, whereas a pattern tracking frequency/cost favors an adaptation-based explanation.

Mandarin provides a typologically informative test case for grammatical impairment because functional structure is primarily realized through particles and nominal functional heads rather than inflectional morphology. Therefore, establishing a Mandarin-appropriate baseline and profiling functional morpheme subtypes in connected speech are necessary for evaluating whether functional reduction in Broca’s aphasia reflects a disproportionate impairment beyond typological baseline patterns. Although prior work has documented content–function asymmetries and has examined selected constructions in Chinese aphasia, connected-speech evidence that systematically profiles multiple lexical and functional morpheme subtypes, and evaluates whether functional reduction is graded across lower versus higher clausal/discourse-related domains, remains limited. Therefore, this study addresses two research questions:

(1) Relative to age- and education-matched healthy controls, do Mandarin-speaking individuals with WAB-classified Broca’s aphasia show a disproportionate reduction of functional morphemes (and a corresponding increase in content-morpheme dominance) in connected speech?

(2) Within connected speech, which lexical-category subtypes and which functional-morpheme subtypes are selectively reduced or relatively preserved in the Broca’s aphasia group, and how are these distributional measures related to individual variability and aphasia severity (AQ)?

## 3 Methodology

### 3.1 Participants

Patients were recruited from the Shanghai Sunshine Rehabilitation Center, and all participants were native monolingual Mandarin speakers. Inclusion criteria included a diagnosis of Broca’s aphasia confirmed through the widely used battery, i.e., Western Aphasia Battery (WAB) (Kertesz, 1982), by clinicians. Exclusion criteria included other neurological conditions or aphasia subtypes (e.g., Wernicke’s aphasia, conduction aphasia, etc.). Aphasia severity was measured using the aphasia quotient (AQ) of WAB, and participants were required to meet the diagnostic criteria for Broca’s aphasia, characterized by non-fluent speech and preserved comprehension. The detailed demographic information for the Broca’s aphasic participants is presented in Table 1. Eighteen participants’ age ranged from 42 to 76 years (mean = 62.22, SD = 10.53) and included eleven male and seven female individuals with diverse educational backgrounds, from primary education to college-level degrees (mean = 10.44 years, SD = 3.36 years). The time post the onset of stroke for the participants ranged from 6 to 16 months (mean = 10.55, SD = 3.05). Besides, the aphasia quotient (AQ) ranged from 27.8 to 68.0 (mean = 51.14, SD = 12.26). All participants were right-handed and had self-reported normal or corrected-to-normal hearing and vision.

**TABLE 1:** Demographic details of participants (patients and healthy controls).

Group	Variable	Mean (SD)	Range
Patients	Age	62.22 (10.53)	42–76
	Years of education	10.44 (3.36)	6–19
	Time post onset (TPO) (months)	10.55 (3.05)	6–16
	Aphasia quotient (AQ)	51.14 (12.26)	27.8–68.0
Healthy controls	Age	62.28 (8.55)	45–76
	Years of education	10.78 (2.96)	6–18

In addition to the aphasia cohort, an age-matched (M = 62.28, SD = 8.55) and education-matched healthy control group (Healthy Control, *n* = 18) was recruited to provide a typologically appropriate baseline for Mandarin picture-description discourse. Demographic information for the healthy control group is also summarized in Table 1 (Healthy controls). Healthy controls (HC) were recruited via community outreach and local advertisements and were screened through a brief health-history interview. Inclusion criteria required native monolingual Mandarin background and normal or corrected-to-normal hearing and vision. Exclusion criteria included any self-reported history of stroke, traumatic brain injury, neurodegenerative disease, psychiatric illness, developmental language/learning disorders, or current medication known to affect cognition. The HC group was recruited to match the patient group at the group level on age and years of education, and all HC participants completed the same WAB picnic-scene picture-description task under the same elicitation and recording protocol. This within-study control baseline was incorporated to strengthen the interpretability of the content-function distribution in Mandarin.

### 3.2 Task

The picture description task of the Spontaneous Speech section of the Mandarin translation of the Western Aphasia Battery was adopted, in which the patients were required to describe what happens in a line drawing of a picnic scene. For patients with limited output, clinicians provided additional prompts to encourage more extensive speech production. As Kertesz (1982) suggests, this section is designed to elicit conversational speech from a patient to measure his/her function communication, information content, speech fluency, lexical access, paraphasias, and grammatical competence. The Mandarin translation of the Western Aphasia Battery has been validated in previous studies (e.g., Qi et al., 2025).

To ensure that each discourse sample provided sufficient material for lexical-category profiling, we set an *a priori* minimum-length criterion of at least 25 intelligible morpheme tokens in the picnic-scene description. If an initial attempt yielded fewer than 25 intelligible morphemes, the clinician delivered standardized neutral prompts (e.g., “Please describe more details about what you see”) up to three times.

### 3.3 Procedure and coding

Written informed consent was obtained from each participant or their legal guardian. Picture-description responses were digitally recorded by trained clinicians following a standardized administration protocol. Recordings were orthographically transcribed and then tokenized at the morpheme level to match Mandarin’s largely isolating morphology. In morpheme segmentation, lexical roots (e.g., nouns, verbs, adjectives) were treated as separate morpheme tokens, while grammatical morphemes/particles (e.g., aspect markers, sentence-final particles, complementizers) were segmented and counted as independent tokens. For example, in verb-aspect sequences, the verb root (e.g., *chi* “eat”) was counted as a content morpheme, whereas the aspect marker (e.g., *le/zhe/guo/zai*) was counted as a function morpheme. Filled pauses, laughter, and unintelligible phonation without recoverable lexical content were excluded from lexical-category coding.

Each morpheme token was coded as either a content or a function item and further classified into subtypes following Huang et al. (2009). Content morphemes included nouns, verbs, adjectives, adverbs, localizers, and numerals, which originate in the lexical layer and carry semantic meaning. Function morphemes included prepositions (e.g., zai, yu, dao, wang), determiners (e.g., de, zhe ge, na ge, zhe xie, na xie), quantifiers (e.g., renhe, dou), pronouns, classifiers (e.g., ge, zhi, tou, tiao, tai, dong), complementizers/particles (e.g., ba, ne, ma, le), aspect markers (e.g., zhe, le, guo, zai), tense/future markers (e.g., jiang, hui), topic/focus markers (e.g., a, ya, ne; cai, jiu), and negators (e.g., bu, mei), which primarily encode grammatical relations.

### 3.4 Data analysis

All analyses were conducted on valid morpheme tokens. Following the WAB guidelines (Kertesz, 1982), neologisms were excluded from both the numerator and the denominator of all measures. Utterances consisting solely of interjections or unintelligible phonation were excluded from lexical-category coding. For each participant, we computed the number of content morphemes (C) and function morphemes (F) and derived a content-to-function ratio (C/F) as the primary dependent measure, to avoid redundancy inherent in complementary percentages. Because one participant produced zero function morphemes (*F* = 0), the ratio is undefined for that case; this participant was retained in descriptive figures but excluded from inferential analyses involving the ratio (patient-group analyses involving C/F: *n* = 17). Group differences were evaluated on per-participant C/F ratios (patients vs. healthy controls). Within the patient group, associations between C/F and participant variables (AQ, TPO, age, and years of education) were assessed using Pearson correlations (df = *n*−2). Descriptive subtype results are reported as proportions for comparability with prior literature.

## 4 Results

### 4.1 Content words vs. function words

#### 4.1.1 Overall results

**FIGURE 1:**
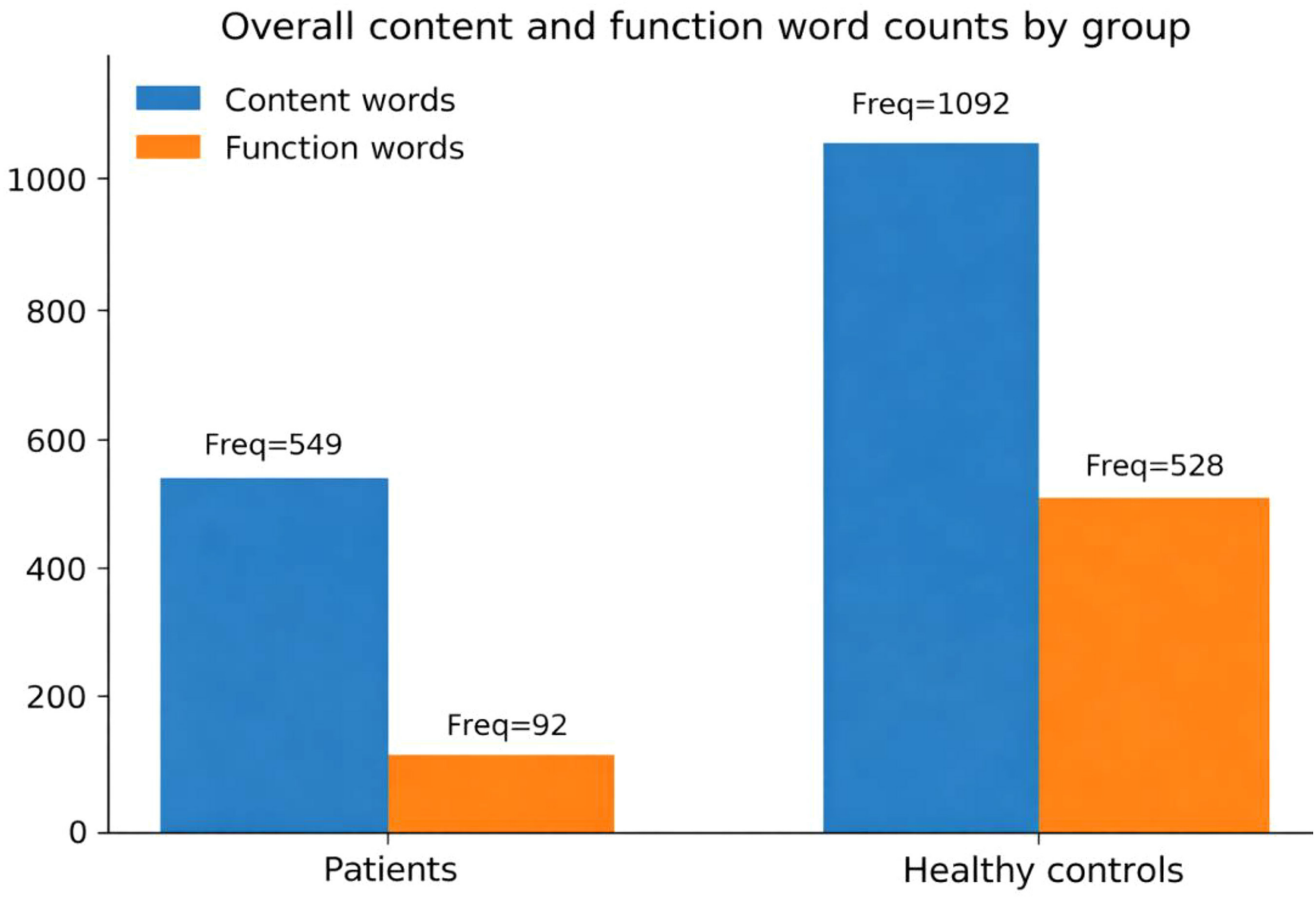
Overall raw token counts of content and function words produced by patients and healthy controls.

The patients’ and healthy control’s usage patterns of content and function words are presented in Figure 1.

The overall distributions of content and function words in the patient and healthy control groups are summarized in Figure 1 using raw token counts. At the token level, patients produced 549 content-word tokens and 92 function-word tokens, whereas healthy controls produced 1,092 content-word tokens and 528 function-word tokens, indicating substantially richer functional scaffolding in neurologically healthy Mandarin discourse. Expressed as a content-to-function ratio (content/function), the patient group showed a markedly larger imbalance (549/92 = 5.97) than the control group (1,092/528 = 2.07). Consistent with this aggregate pattern, per-participant proportions in the patient cohort indicated that content words constituted a mean of 85.48% (range = 61.3%–100%), whereas function words constituted a mean of 14.54% (range = 0%–38.7%), revealing pronounced inter-individual variability including individuals with zero function-word output. In subsequent inferential analyses, we therefore use the content-to-function ratio as the primary dependent measure to avoid redundancy inherent in complementary percentages.

As shown in Table 2, Pearson correlation analyses were conducted using the content-to-function ratio (content/function) as the dependent variable. Aphasia severity was indexed by the aphasia quotient (AQ; higher scores indicate milder aphasia). The results revealed a significant negative correlation between AQ and the content-to-function ratio, *r*(15) = −0.513, *p* = 0.035. This indicates that patients with higher AQ scores (i.e., less severe aphasia) exhibited a more balanced distribution between content and function words, whereas patients with more severe aphasia showed a stronger bias toward content-word production relative to function words.

**TABLE 2:** Correlation coefficients between participant variables and the content-to-function ratio (content/function).

Group	Predictor	*r*	*P*
Patients	Aphasia quotient (AQ)	–0.513	0.035
	Time post onset (TPO, months)	0.249	0.336
	Age	–0.093	0.724
	Years of education	–0.227	0.380
Healthy controls	Age	–	–
	Years of education	–	–

One patient with *F* = 0 was excluded from ratio-based correlations (*n* = 17).

No significant correlations were observed between the content-to-function ratio and time post onset (TPO), age, or years of education (all *p* > 0.3), suggesting that the observed imbalance in lexical-category production is not reliably modulated by these demographic or temporal factors within the present sample.

**FIGURE 2:**
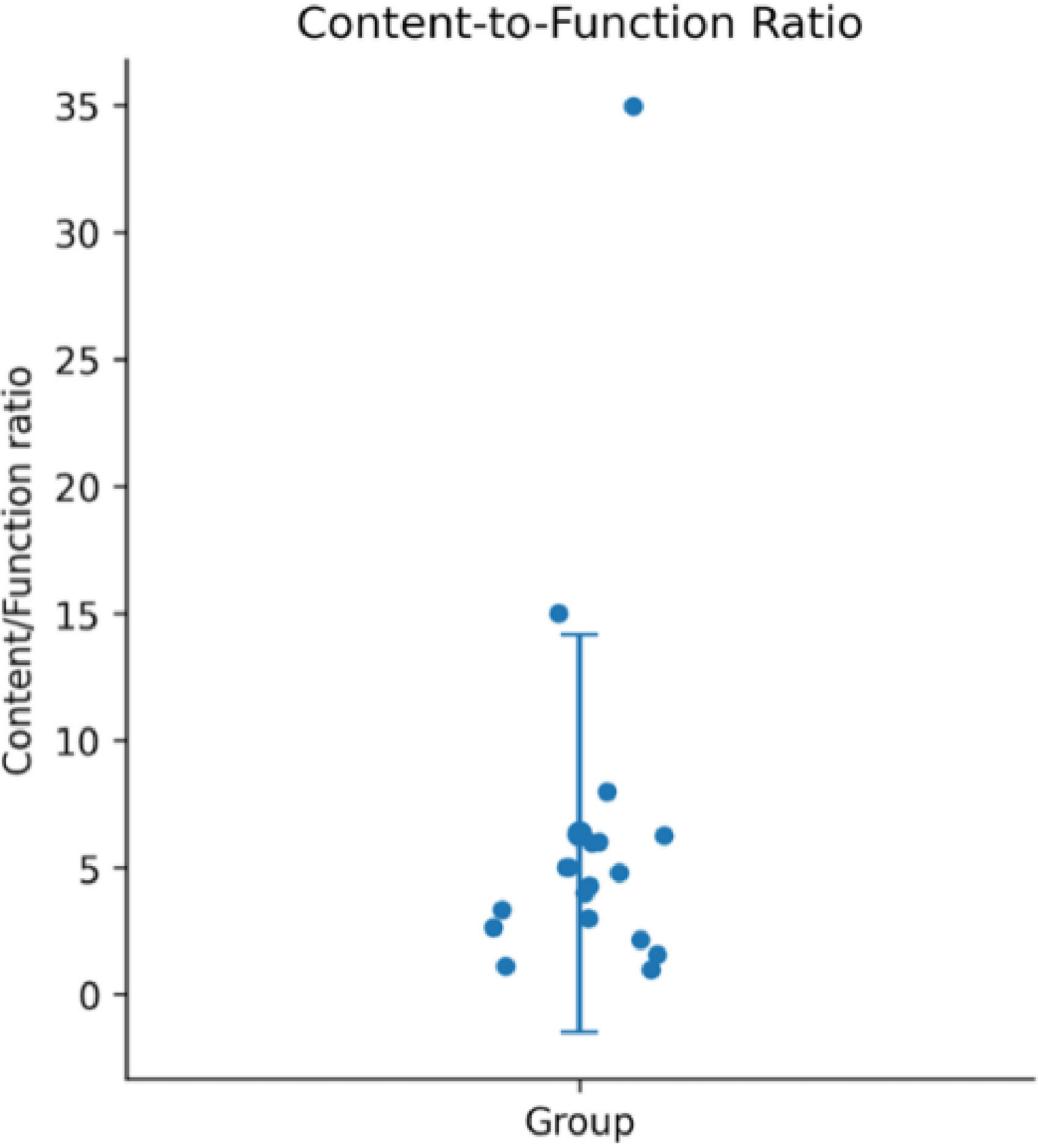
Content-to-function ratio (content/function) across participants. Points represent individual participants; the central marker indicates the mean and error bars represent standard deviation (SD).

As Figure 2 illustrates, the content-to-function ratio exhibited substantial variability across participants. While the majority of individuals clustered within a relatively moderate range, a small number of participants showed markedly elevated ratios, indicating a disproportionate reliance on content words relative to function words. The presence of these high-value outliers contributed to an increased standard deviation, reflecting considerable inter-individual differences in lexical distribution. Overall, the data suggest that although a general tendency toward content-heavy production is observable in Mandarin-speaking individuals with Broca’s aphasia, the extent of this imbalance varies widely across patients. This variability underscores the heterogeneity of lexical production profiles within the aphasic population and suggests that the content-function dissociation is not uniform across individuals.

#### 4.1.2 Individual analysis

**FIGURE 3:**
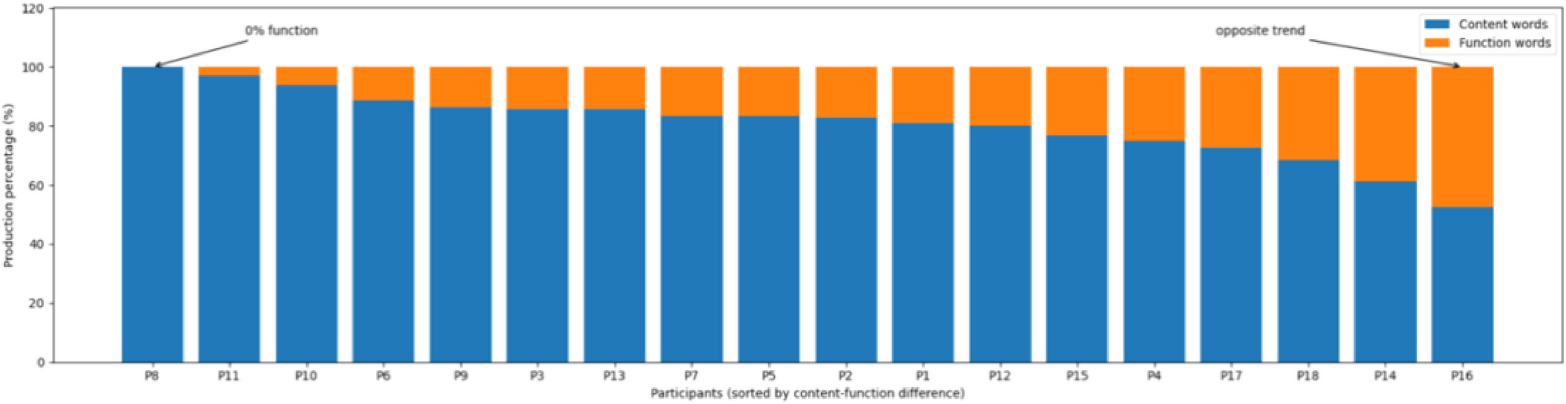
Individual profiles of content- and function-word production.

To further illustrate individual performance in the production of content and function words, participant-level results are presented in Figure 3. The black bars represent the percentage of content words and the gray bars represent the percentage of function words; the horizontal axis lists participants (P1–P18) and the vertical axis indicates production percentages. As shown in Figure 3, most participants produced substantially higher proportions of content words than function words. For instance, P3, P6, and P10 showed high content-word production (approximately 85% to over 93%), while producing much lower proportions of function words, reflecting a general tendency toward content-word dominance at the individual level.

However, several participants exhibited atypical profiles that warrant additional follow-up analyses. In particular, P8 produced no function words at all, relying entirely on content words (100% content words). In contrast, although P11 also demonstrated an extremely high content-word proportion (97.22%), a small proportion of function words was still present (2.78%), indicating that complete omission of function words was limited to a single case in the current dataset.

In addition, P16 produced 52.63% content words and 47.37% function words, representing the lowest content-word proportion and the highest function-word proportion in the sample. This yields a content–function difference of 5.26 percentage points, which is markedly smaller than the group-level tendency (M content ≈ 81%–85% vs. M function ≈ 15%–19%, depending on aggregation method), indicating a substantially more balanced lexical distribution relative to other participants.

To further interpret these atypical quantitative profiles, we conducted a qualitative inspection of the WAB picture-description transcripts for P8, P11, and P16. For P8 (0% function-word production; Figure 3), the transcript was characterized by utterances consisting predominantly of isolated lexical items and short sequences of content words, with minimal evidence of clause-level grammatical scaffolding. Function morphemes that typically encode grammatical relations in Mandarin, such as structural particles (e.g., de-type markers) and aspectual morphology (e.g., −le, −zhe, −guo), were absent throughout the sample or restricted to rare, non-productive occurrences. As a result, propositional relations (e.g., temporality, location, possession, argument structure) were conveyed primarily via lexical choice rather than overt functional marking, yielding sentences that were short and weakly integrated syntactically. This qualitative profile suggests a pattern consistent with substantial reduction of functional structure, even when lexical retrieval remains relatively available.

For P11, although a small number of function words was produced, the overall profile remained highly content-dominant. Function elements were sparse and did not contribute consistently to syntactic structuring, indicating that grammatical encoding was still severely constrained despite not being entirely absent. By contrast, P16 showed a qualitatively different deviation from the group pattern. The elevated function-word proportion in P16 was not associated with richer syntactic integration; instead, function-word tokens clustered in a limited set of high-frequency closed-class forms across the transcript. Many of these function elements appeared to serve discourse-supporting or formulaic roles. When these closed-class forms were set aside, the remaining propositional content was still carried mainly by content words, and clause structure remained relatively simple.

Taken together, these case-level observations clarify why P8, P11, and P16 diverge from the group-level tendency and underscore the value of qualitative evidence in an underpowered sample: extreme omission of function morphemes (P8), near-complete reduction (P11), and formula-driven inflation of function-word counts (P16) represent distinct routes to atypical content–function distributions.

### 4.2 Subtypes of each classification

The results for the content-word subtypes are summarized in Table 3. Overall, the distribution shows a clear lexical pattern in patients’ content-word production. Nouns overwhelmingly dominated the patients’ speech, far surpassing all other categories. However, the verb contrast did not reach statistical significance, so we treat it as descriptive. The remaining subcategories were rare. The relatively large SD for nouns indicates substantial between-participant variability in noun production, suggesting uneven reliance on nouns across individuals. Numerals showed low means but relatively large SDs, indicating that their production was uneven across participants rather than consistently present in all samples. Adjectives, localizers, and adverbs occurred at very low rates overall, suggesting that these categories were particularly difficult for patients to produce and may be especially vulnerable in post-stroke aphasia.

**TABLE 3:** Results of subtypes of content words (PWA and HC).

Subcategories	PWA mean (SD)	HC mean (SD)
Nouns	63.02% (0.214)	32.04% (0.026)
Verbs	11.73% (0.101)	14.99% (0.030)
Numerals	3.21% (0.065)	2.67% (0.015)
Adjectives	1.49% (0.024)	5.34% (0.021)
Localizers	1.18% (0.026)	3.73% (0.016)
Adverbs	0.53% (0.015)	3.10% (0.012)

In addition, the content-word subtype profile for the healthy control group is also summarized in Table 3. Overall, healthy speakers showed a more distributed lexical profile than the Broca’s aphasia group: nouns remained the largest content category, but verbs were produced at substantially higher rates, and modifiers and relational categories were consistently present, including adjectives, adverbs, and localizers. Compared with patients, the smaller standard deviations across subtypes indicate reduced between-speaker variability in healthy discourse production, consistent with stable access to event structure and descriptive elaboration in neurologically intact speakers.

To statistically evaluate group differences across content-word subtypes, we conducted independent-samples *t*-tests on per-participant proportions (patients vs. healthy controls; *n* = 18 per group). Independent-samples *t*-tests were conducted on per-participant proportions across six content-word subtypes. With Bonferroni correction (α = 0.05/6 = 0.008), patients produced a significantly higher proportion of nouns than healthy controls, *t* = 6.10, *p* < 0.001, whereas healthy controls produced significantly higher proportions of adjectives (*t* = −5.12, *p* < 0.001), localizers (*t* = −3.54, *p* = 0.001), and adverbs (*t* = −5.68, *p* < 0.001). Group differences for verbs and numerals were not significant (ps ≥ 0.204).

Table 4 shows that function-word production in the patients was concentrated in only a few subtypes, while most categories were marginal or absent. The highest mean was observed for determiners, with classifiers as the next most frequent category. The large dispersions for both determiners and classifiers indicate that these forms were not consistently accessible across patients; instead, a small subset of individuals likely contributed disproportionately to their occurrence. Beyond these two categories, frequencies dropped sharply: focus markers and aspect markers appeared infrequently and variably, suggesting only intermittent access to these functional elements. The remaining categories were near floor levels, i.e., pronouns, negation, quantifiers, and complementisers, indicating that they surfaced only sporadically in the dataset. Importantly, tense markers and topic markers were completely absent from all productions. This distribution points to a sharply graded impairment within the functional lexicon: comparatively frequently used closed-class items (especially determiners and classifiers) showed partial preservation with substantial individual variability, whereas categories associated with higher-level clause structure (e.g., complementisers) and discourse/grammatical organization (tense and topic marking) were the most vulnerable.

**TABLE 4:** Results of subtypes of function words (patients and healthy controls).

Subcategories	PWA mean (SD)	HC mean (SD)
Prepositions	4.32% (0.080)	5.51% (0.015)
Determiners	6.66% (0.089)	8.74% (0.017)
Classifiers	3.15% (0.065)	6.33% (0.013)
Focus markers	1.73% (0.044)	2.70% (0.009)
Aspect markers	1.16% (0.021)	4.02% (0.012)
Pronouns	0.75% (0.024)	3.78% (0.010)
Negation	0.52% (0.015)	1.96% (0.008)
Quantifiers	0.29% (0.012)	2.35% (0.008)
Complementisers	0.28% (0.010)	1.17% (0.006)
Tense markers	0	0.40% (0.004)
Topic markers	0	1.18% (0.006)

In addition, Table 4 also presents the function-word subtype distribution in healthy Mandarin picture-description discourse. Function-word production in HC was not restricted to a small set of closed-class items; rather, multiple subtypes were consistently realized. Determiners and classifiers constituted the largest functional categories, reflecting robust nominal functional structure in Mandarin. Importantly, higher-level and discourse-related function types were also systematically present, including focus markers and topic markers, alongside complementisers. Although tense/future marking is generally limited and optional in Mandarin, tense markers were nevertheless observable at low but non-zero rates, consistent with occasional future-oriented modal/auxiliary uses in spontaneous description. Together, this control-group profile establishes a typologically appropriate baseline in which Mandarin functional morphology and particles are available across multiple layers, providing a contrastive reference for interpreting the selective reduction observed in Broca’s aphasia.

Independent-samples *t*-tests were conducted on per-participant proportions across 11 function-word subtypes. With Bonferroni correction (α = 0.05/11 = 0.005), healthy controls produced significantly higher proportions of aspect markers (*t* = −5.02, *p* < 0.001), pronouns (*t* = −4.94, *p* < 0.001), negation (*t* = −3.59, *p* = 0.001), quantifiers (*t* = −6.06, *p* < 0.001), complementisers (*t* = −3.24, *p* = 0.003), tense markers (*t* = −4.24, *p* < 0.001), and topic markers (*t* = −8.34, *p* < 0.001) than patients. Differences in determiners, focus markers, and classifiers were not significant (ps ≥ 0.057).

## 5 Discussion

This Discussion proceeds in three steps. We first interpret the overall content-function imbalance against a Mandarin-appropriate baseline and relate it to structural versus processing-based accounts. We then address subtype-level patterns in the lexical and functional domains with particular attention to hierarchy-based predictions. Finally, we outline typology-appropriate clinical implications for assessment and intervention in Mandarin-speaking Broca’s aphasia.

### 5.1 Content vs. function word production

The present results show a robust content-function asymmetry in Mandarin Broca’s aphasia: across participants, lexical items dominated connected speech output, while functional items were markedly reduced. Interpreting this imbalance in Mandarin requires an explicit typological baseline. Because Mandarin lacks articles and does not express tense/agreement through inflection, the absolute proportion of overt function elements in normal Mandarin discourse is not expected to mirror English by default (Li and Thompson, 1981; Lin, 2006). To establish a typologically appropriate baseline within a tightly controlled design, we recruited an age- and education-matched healthy control group who completed the same WAB picnic-scene picture-description task under the same elicitation, transcription, and coding procedures. The patient–control contrast therefore provides the primary benchmark for interpreting the present results, indicating that the reduction of functional material in the Broca’s aphasia cohort reflects a disproportionate reduction relative to neurologically intact Mandarin discourse rather than a typological property of Mandarin *per se*.

Within a focused generative account, the pattern aligns with the Tree-Pruning Hypothesis when adapted to Mandarin’s functional inventory. The near-floor performance on categories plausibly linked to higher clausal and discourse-related structure, alongside sporadic availability of nominal functional elements such as demonstratives/classifiers, is consistent with a graded vulnerability of functional projections (Friedmann and Grodzinsky, 1997). Crucially, in a tenseless language, missing tense markers cannot be taken as the key diagnostic; rather, the relevant prediction concerns broader clausal and discourse-related functional scaffolding. From this perspective, the scarcity of particles and functional devices that support aspectual anchoring, clause-typing, and discourse packaging is informative precisely because these are among the primary ways Mandarin encodes grammatical relations without inflection (Huang et al., 2009).

At the same time, the magnitude and heterogeneity of functional reduction across individuals are naturally captured by a resource-limitation/adaptation perspective. In spontaneous production, function elements contribute little lexical meaning but impose planning and integration demands: they require the speaker to maintain a structural plan, map argument structure onto linear order, and deploy appropriate particles in real time. Under constrained processing resources, speakers may therefore adopt a “lexical survival” strategy, producing content-heavy sequences that convey core semantic roles while leaving grammatical relations underspecified. This helps explain why some individuals produced virtually no function items at all, whereas others showed inflated counts of a narrow subset of high-frequency closed-class forms. Importantly, this adaptation mechanism does not replace the structural account; it provides a production-level explanation for why a structurally vulnerable functional domain becomes especially fragile in connected speech.

The observed reduction of grammatical markers, particularly complementizers and topic particles, supports the Tree-Pruning Hypothesis by indicating vulnerability in higher projections. However, the partial preservation of classifiers and determiners, especially those with high frequency and low morphosyntactic burden, suggests that processing-based adaptation also plays a role. We interpret the observed distribution of functional elements cautiously, given that frequency and distributional factors may constrain strong theoretical adjudication between structural and functional accounts. The present findings are therefore treated as descriptive evidence about content-function imbalance in Mandarin discourse. Furthermore, Mandarin’s analytic structure and lack of overt tense morphology likely amplify these effects, as grammatical information is often encoded through standalone particles rather than inflections. Together, the results demonstrate that while structural accounts like TPH explain the vertical degradation of syntax, adaptation theory captures the horizontal selectivity across functional elements. The integration of both theories, anchored in the typological properties of Mandarin, offers a nuanced understanding of grammatical deficits in Broca’s aphasia.

Importantly, the individual-level analyses reported in Section “4.1.2 Individual analysis” provide converging evidence that the group-level content-function imbalance reflects clinically meaningful heterogeneity. Specifically, participants with near-absent functional material (e.g., P8 and P11) exhibited highly fragmentary, content-heavy output that aligns with severe disruption in accessing functional structure, whereas an atypical quantitative profile (e.g., P14) suggests that an apparently high proportion of functional morphemes may be supported by a narrow and formulaic subset rather than productive deployment of the broader functional inventory. Taken together, these profiles strengthen the interpretation that reduced functional material in the present cohort represents a disproportionate reduction associated with non-fluent aphasia, while also indicating that the magnitude and surface manifestation of this reduction vary substantially across individuals.

By “clinically meaningful heterogeneity,” we refer to systematic inter-individual variability in the content-to-function ratio that bears on functional-morpheme accessibility. The ratio covaries with aphasia severity (AQ), it can help clinicians profile patients who show disproportionate difficulty with function words despite producing substantial lexical content. Such profiling may complement global severity indices with a discourse-based functional-access marker and may guide intervention by prioritizing function-word scaffolding and sentence-frame practice for patients with markedly elevated ratios.

### 5.2 Distribution across subcategories

A strong test of TPH would require demonstrating individual-level dissociations. Group-level proportional data in the present design cannot establish such dissociations; therefore, we treat the present discussion as hypothesis-generating. Analysis of subtypes revealed further differentiation within each word category. Among content-word subtypes, nouns accounted for the largest share of content morphemes in the Broca’s aphasia group. Although the verb proportion was numerically lower than in healthy controls, the between-group comparison for verbs did not reach statistical significance in the present sample (and therefore does not support a verb-specific group difference). In addition, subtype proportions within the content domain are compositional (i.e., increases in one subtype necessarily reduce the remaining subtypes), such that noun and verb proportions are not statistically independent. For these reasons, we treat the observed noun–verb numerical contrast as descriptive and refrain from providing a functional or mechanistic interpretation of “verb impairment” based on these data. As background, prior work has reported noun–verb dissociations in aphasia under specific task demands and languages (Gleason et al., 1980; Breedin et al., 1998; Rezaii et al., 2023; Chen and Bates, 1998; Luzzatti et al., 2002; Druks, 2002; Hillis and Caramazza, 1995; Li and Kiran, 2024), and has discussed these patterns in relation to semantic concreteness and differences in syntactic/argument-structure demands associated with verbs (Plaut and Shallice, 1993; Thompson et al., 1997; Luzzatti et al., 2002; Bastiaanse et al., 2002). However, because the present study did not detect a significant between-group difference in verb proportions (and because proportion-based subtype contrasts are not independent), we do not treat these accounts as explanations of the current findings; rather, establishing a verb-specific deficit in connected speech would require designs that directly test verb retrieval while controlling compositional and frequency-related constraints.

Consistent with the subgroup statistics in Section “4.2 Subtypes of each classification,” between-group tests confirmed that patients produced a significantly higher proportion of nouns, whereas healthy controls produced significantly higher proportions of modifiers (adjectives/adverbs) and localizers, indicating reduced descriptive elaboration and spatial/relational encoding in aphasic discourse; notably, these categories were infrequent overall, reflecting minimal descriptive/relational detail in the discourse samples. Formulaic expressions often occur in some patients, supporting prior claims that such utterances are preserved because of their fixed structure in memory and right-hemisphere mediation (Code, 2005; Torrington Eaton and Burrowes, 2022).

Function words showed more prominent subtype variability. Topic markers, complementisers, and tense markers, associated with high projections, were almost or entirely absent, whereas focus markers showed limited preservation, probably due to some patients’ individual preference in production. Aspect and negation, involving lower syntactic nodes, were also rarely produced. Pronouns and quantifiers, which are situated even lower, appeared occasionally. Crucially, the between-group comparisons showed that the aphasia group was significantly reduced across multiple clausal/discourse-related and operator-like subtypes (e.g., aspect markers, negation, pronouns, quantifiers, complementisers, tense markers, and topic markers), strengthening the interpretation of selective vulnerability within the functional inventory beyond mere descriptive differences. Pronoun production was sparse in the Broca’s aphasia group and significantly reduced relative to healthy controls, consistent with a broader reduction of closed-class functional material in connected speech.

In addition, determiners and classifiers were occasionally produced, suggesting partial access within the DP domain; descriptively, a small subset of highly frequent nominal functional elements (e.g., classifier-related material) appeared in some patient transcripts, suggesting partial access to residual DP resources. From the generative grammar perspective, these patterns reinforce the severe impairment of higher syntactic categories and relatively milder damage to the lower ones, which is consistent with the aforementioned Tree-Pruning Hypothesis (Friedmann and Grodzinsky, 1997). This supports the notion that semantic and grammatical processes can be independently impaired (Tzeng et al., 1991), reinforcing the dual-route model in aphasic language production (Rosenberg et al., 1985).

### 5.3 Clinical implications

A central clinical implication of the present study is that, in Mandarin, reduced grammatical scaffolding in non-fluent aphasia is most transparently captured by function-morpheme profiles in connected speech. Cross-linguistically, function-morpheme reduction is a well-attested characteristic of non-fluent aphasia; Mandarin does not differ in whether functional material is vulnerable, but rather in how functional structure is overtly realized. Because Mandarin relies heavily on particles and nominal functional heads (e.g., classifiers) to package grammatical relations, a reduction of such material can yield content-heavy utterances with weakened event anchoring and discourse organization (Huang et al., 2009; Li and Thompson, 1981; Lin, 2006).

Subtype patterns further suggest that assessment and intervention targets should be typology-appropriate. In the present dataset, nominal functional material (especially determiner-/classifier-related categories) showed partial availability with substantial individual variability, whereas multiple clausal/discourse-related functional types were near-floor. Clinically, this motivates a staged approach that leverages residual nominal resources to rebuild more structured utterance frames, while also incorporating explicit training on functional devices that Mandarin uses to encode aspectual anchoring and clause/discourse packaging.

Finally, the robust association between aphasia severity (AQ) and the content–function balance indicates that function-morpheme measures can serve as sensitive indices of recovery in Mandarin connected speech: milder aphasia is associated with greater access to functional material, whereas more severe aphasia yields stronger reliance on lexical content. This suggests that tracking function-morpheme profiles over time may be useful both for outcome measurement and for individualizing intervention priorities.

## 6 Conclusion

In summary, this study conducted a detailed analysis of the lexical categories in the spoken output of Mandarin-speaking Broca’s aphasic patients, confirming the overall trend of content-word dominance in aphasia while revealing heterogeneity within subcategories underlying these differences. Regarding content words, the classic pattern of relatively preserved nouns and verbs showed a numerically lower proportion; however, the verb difference was not statistically significant in the present sample in Mandarin aphasia. As for function words, the production of different grammatical elements was uneven, with certain categories proving particularly vulnerable. Besides, it is revealed that aphasia severity was a significant predictor of lexical category production, that is, patients with milder aphasia predict increased use of function words and decreased reliance on content words. In contrast, factors such as age, and years of education showed no significant associations with the production of either word type. These findings provide evidence for understanding the differential impairment mechanisms affecting lexical and grammatical elements in aphasia, with aphasia severity, not age, years of education, or time post onset, as the key influencing factor.

From a clinical rehabilitation standpoint, this study underscores the importance of assessing and targeting specific word classes, i.e., intervention programs should be customized to each patient’s deficits. For Mandarin, a language heavily reliant on function words to convey grammatical relations, these results offer empirical support for designing targeted therapy strategies, such as specialized training in classifiers and aspect markers, and sentence frame construction exercises, that can strengthen the precision and effectiveness of aphasia rehabilitation, ultimately enhancing patients’ practical communication abilities.

## Data Availability

The original contributions presented in this study are included in this article/supplementary material, further inquiries can be directed to the corresponding author.
